# The Effects of Dietary Protein and Polysaccharide Fortification on Disease

**DOI:** 10.3390/nu15194137

**Published:** 2023-09-25

**Authors:** Junjie Luo, Yongting Luo

**Affiliations:** Department of Nutrition and Health, Beijing Advanced Innovation Center for Food Nutrition and Human Health, China Agricultural University, Beijing 100193, China

Proteins and polysaccharides are versatile natural macromolecules that are ubiquitous in nature, and a tailored diet that is fortified with them has been developed to ameliorate a wide array of diseases. The goal of this Special Issue of *Nutrients*, entitled “Effects of dietary protein and polysaccharide fortification on disease”, is to collect a series of manuscripts that cover all beneficial aspects related to proteins and polysaccharides, especially those that examine the treatment of various disorders and their complications.

This Special Issue contains 17 papers, including nine articles, three reviews, three systematic reviews, one comment, and one reply. The topics covered include the following: the burden of protein–energy malnutrition (one paper); a case study of 18 adults on low-protein diets (one paper); the health benefits of egg protein (one paper); proteins in breast milk during different lactation periods (one paper); protein intake and frailty in older adults (three papers); protein intake and the risk of hypertension (one paper); casein hydrolysate on cardiovascular risk factors (one paper); oral lactoferrin on iron-deficiency anemia (one paper); novel soy peptide and osteoblast differentiation (one paper); different protein sources and their ability to enhance the uptake of brown adipocytes (one paper); polysaccharides in anti-aging or liver diseases (two papers); β-glucan and its ability to protect against doxorubicin-induced cardiotoxicity or improve lipid metabolism (two papers); and different carbohydrate–fat ratios in enteral nutrition on metabolic pattern and organ damage (one paper).

Malnutrition occurs when the nutritional requirements for growth (proteins and polysaccharides) are not met within the context of either undernutrition or overnutrition. This condition represents the largest single contributor to disease development, which affects the quality of life in both developing and developed countries. For this reason, one key objective of the WHO Global Action Plan for the Prevention and Control of Noncommunicable Diseases 2013–2020 was to promote healthy diets using nutritional intervention, with particular emphasis placed on maternal, infant, and early childhood nutrition. In this Special Issue, Xu Zhang et al. reported that protein–energy malnutrition is a relatively serious disease burden in the world, especially among children and the elderly [1]. Moreover, data from IEIPM (inborn errors of intermediary protein metabolism) adults on low-protein diets confirmed that the trend of overweight and obesity in children is increasing despite low energy intakes, suggesting that low protein intake may contribute to increased fat mass [2]. These studies support the notion that effective nutritional prevention measures should be strengthened to continuously improve public health conditions. In fact, in this Special Issue, egg protein was evaluated to be an excellent source of essential amino acids that can reduce malnutrition in underdeveloped countries, possibly increase the height of children, and prevent kwashiorkor [3]. Furthermore, Yifan Zhang et al. analyzed the protein composition of breast milk and provided necessary support for the design and production of infant formula [4]. On the other hand, Coelho-Junior et al. demonstrated that, in older adults, protein intake was not significantly associated with frailty, but frail older adults consumed significantly less animal protein than their healthy counterparts [5]. Interestingly, William B. Grant believed that Coelho-Junior et al. omitted the most important reason for this finding, which is that animal products are a great source of vitamin D, and vitamin D can reduce the risk of frailty [6]. However, Coelho-Junior et al. disagreed with Grant that vitamin D is the main mediator in the relationship between animal products and frailty [7]. While conclusions are yet to be drawn, it is clear that these topics should be discussed further.

Insufficient dietary protein intake has also been observed to be closely associated with the risk of cardiovascular diseases. Jingjing He et al. indicated that there is a beneficial association between animal, plant, and total protein consumption and hypertension risk at lower intake levels, while excessive intake of plant or total protein may increase the risk of hypertension in the Chinese population [8]. Shuaishuai Zhou et al. found that casein hydrolysate supplementation was shown to lower blood pressure without affecting blood lipids or glycemic status [9]. The intake of specific proteins is closely associated not only with cardiovascular risk but also with the fight against anemia, osteoporosis, and obesity. Xiya Zhao et al. showed that lactoferrin was a superior supplement to ferrous sulfate regarding the improvement in serum iron parameters and hemoglobin levels, and the anti-inflammatory effect of lactoferrin may be the underlying mechanism [10]. Kuaitian Wang et al. indicated the novel soy peptide CBP (calcium-binding peptide) could stimulate osteoblast differentiation and bone mineralization, further highlighting the important potential of CBP in the treatment of osteoporosis [11]. By evaluating the association between different dietary protein sources and brown adipose tissue activity (BAT), Katarzyna Maliszewska et al. illustrated that different macronutrient consumptions may serve as a new means of regulating BAT activity leading to weight loss [12].

While much attention is given to proteins, both proteins and polysaccharides play a vital role in maintaining health and fighting diseases. Xinlu Guo et al. elucidated the anti-aging mechanisms of polysaccharides involving oxidative damage, age-related genes and pathways, immune modulation, and telomere attrition [13]. Yijing Ren et al. summarized the current understanding of the mechanisms of ferroptosis and liver injury, as well as the compelling preclinical evidence of polysaccharides for the treatment of liver disease [14]. Of note, β-glucan, which may be considered a “star polysaccharide”, can both prevent doxorubicin-induced cardiotoxicity by increasing antioxidant capacity and inhibiting oxidative stress [15] and reduce obesity by improving blood lipid levels and accelerating lipidolysis [16]. Lastly, Yongjun Yang et al. explored the optimal carbohydrate–fat ratio to provide an experimental basis for optimizing the nutritional formula for burn patients [17].

In summary, proteins and polysaccharides serve as bioactive nutrients that act as antioxidants or active factors, reduce inflammation and oxidative stress, improve mitochondrial dysfunction, and modulate epigenetic modifications. According to the findings presented in this Special Issue, specific proteins and polysaccharides are beneficial for human beings, which attests to the concepts of “food as medicine” and “preventive treatment” and that these nutrients can be part of special dietary guidance for the clinical treatment of diseases.
